# Sodium acetate/sodium butyrate alleviates lipopolysaccharide-induced diarrhea in mice *via* regulating the gut microbiota, inflammatory cytokines, antioxidant levels, and NLRP3/Caspase-1 signaling

**DOI:** 10.3389/fmicb.2022.1036042

**Published:** 2022-10-28

**Authors:** Xiushuang Chen, Qinghui Kong, Xiaoxiao Zhao, Chenxi Zhao, Pin Hao, Irfan Irshad, Hongjun Lei, Muhammad Fakhar-e-Alam Kulyar, Zeeshan Ahmad Bhutta, Hassan Ashfaq, Qiang Sha, Kun Li, Yi Wu

**Affiliations:** ^1^Institute of Traditional Chinese Veterinary Medicine, College of Veterinary Medicine, Nanjing Agricultural University, Nanjing, China; ^2^MOE Joint International Research Laboratory of Animal Health and Food Safety, College of Veterinary Medicine, Nanjing Agricultural University, Nanjing, China; ^3^College of Animal Science, Tibet Agricultural and Animal Husbandry University, Nyingchi, China; ^4^Institute of Continuing Education and Extension, University of Veterinary Animal Sciences, Lahore, Pakistan; ^5^Department of Animal Nutrition and Feed Science, College of Animal Science and Technology, Huazhong Agricultural University, Wuhan, China; ^6^College of Veterinary Medicine, Huazhong Agricultural University, Wuhan, China; ^7^College of Veterinary Medicine, Chungbuk National University, Cheongju, Chungbuk, South Korea; ^8^Jiangsu Key Laboratory of Pesticide Science, Department of Chemistry, College of Sciences, Nanjing Agricultural University, Nanjing, China

**Keywords:** sodium acetate, sodium butyrate, LPS, diarrhea, microbiota

## Abstract

Diarrhea is a word-widely severe disease coupled with gastrointestinal dysfunction, especially in cattle causing huge economic losses. However, the effects of currently implemented measures are still not enough to prevent diarrhea. Previously we found that dropped short-chain fatty acids in diarrhea yaks, and butyrate is commonly known to be related to the epithelial barrier function and intestinal inflammation. However, it is still unknown whether sodium acetate/sodium butyrate could alleviate diarrhea in animals. The present study is carried out to explore the potential effects of sodium acetate/sodium butyrate on lipopolysaccharide-induced diarrhea in mice. Fifty ICR mice were randomly divided into control (C), LPS-induced (L), and sodium acetate/sodium butyrate (D, B, A)-treated groups. Serum and intestine samples were collected to examine inflammatory cytokines, antioxidant levels, relative gene expressions *via* real-time PCR assay, and gut microbiota changes through high-throughput sequencing. Results indicated that LPS decreased the villus height (*p* < 0.0001), increased the crypt depth (*p* < 0.05), and lowered the villus height to crypt depth ratio (*p* < 0.0001), while sodium acetate/sodium butyrate supplementation caused a significant increase in the villus height (*p* < 0.001), decrease in the crypt depth (*p* < 0.01), and increase in the villus height to crypt depth ratio (p < 0.001), especially. In mice treated with LPS, it was found that the serum level of IL-1β, TNF-α (*p* < 0.001), and MDA (*p* < 0.01) was significantly higher; however, sodium acetate/sodium butyrate supplementation significantly reduced IL-1β (*p* < 0.001), TNF-α (*p* < 0.01), and MDA (*p* < 0.01), respectively. A total of 19 genera were detected among mouse groups; LPS challenge decreased the abundance of *Lactobacillus, unidentified F16, unidentified_S24-7, Adlercreutzia, Ruminococcus, unclassified Pseudomonadales, [Ruminococcus], Acetobacter, cc 1, Rhodococcus, unclassified Comamonadaceae, Faecalibacterium*, and *Cupriavidus*, while increased *Shigella, Rhodococcus, unclassified Comamonadaceae*, and *unclassified Pseudomonadales* in group L. Interestingly, sodium acetate/sodium butyrate supplementation increased *Lactobacillus, unidentified F16, Adlercreutzia, Ruminococcus, [Ruminococcus], unidentified F16, cc 115, Acetobacter, Faecalibacterium*, and *Cupriavidus*, while decreased *Shigella, unclassified Enterobacteriaceae, unclassified Pseudomonadales, Rhodococcus*, and *unclassified Comamonadaceae*. LPS treatment upregulated the expressions of ZO-1 (*p* < 0.01) and NLRP3 (*p* < 0.0001) genes in mice; however, sodium acetate/sodium butyrate solution supplementation downregulated the expressions of ZO-1 (*p* < 0.05) and NLRP3 (*p* < 0.05) genes in treated mice. Also, the LPS challenge clearly downregulated the expression of Occludin (*p* < 0.001), Claudin (*p* < 0.0001), and Caspase-1 (*p* < 0.0001) genes, while sodium acetate/sodium butyrate solution supplementation upregulated those gene expressions in treated groups. The present study revealed that sodium acetate/sodium butyrate supplementation alleviated LPS-induced diarrhea in mice *via* enriching beneficial bacterium and decreasing pathogens, which could regulate oxidative damages and inflammatory responses *via* NLRP3/Caspase-1 signaling. The current results may give insights into the prevention and treatment of diarrhea.

## Introduction

Diarrhea is a severe disease coupled with gastrointestinal dysfunction that has a global impact on fertility rate, milk production, and immunity in livestock (Coura et al., 2015; Li et al., 2022). Nowadays diarrhea in dairy cows and yak is very serious. It has a high incidence rate, especially neonatal calf diarrhea is found usually with high morbidity and mortality, causing considerable economic damage to the industry due to the heavy treatment expenses and impairments in the growth of animal (Coura et al., 2015; Schmoeller et al., 2021). Despite measures such as improved hygiene and scientific feeding management with the use of extensive drugs, this disease, i.e., diarrhea, remains serious (Li et al., 2022). The imbalance in gut microbiota was commonly recognized as the primary cause of diarrhea (Schmoeller et al., 2021), and many studies found changed intestine microbiota in diarrhea of cattle (Chuang et al., 2022; Coelho et al., 2022; Li et al., 2022; Liu J. et al., 2022).

Gut microflora is composed of millions of microorganisms that contribute remarkably to physiological processes, i.e., functions of nutrition absorption, metabolism, and immunity of the host by producing various metabolites (Wei et al., 2020). The anaerobic bacterial fermented short-chain fatty acids (SCFAs) are six carbon-containing fatty acids in the gut (Du et al., 2021). Acetate and butyrate are mainly produced through bacterial catabolism of dietary fibers in the host colon (Ezzine et al., 2022), which are a primary source of energy for colonic epithelial cells (Fu et al., 2019). Previous studies found that acetate could promote small intestinal barrier function in mice (Yosi et al., 2022) and regulate IgA reactivity (Takeuchi et al., 2021). As an important short-chain fatty acid, butyrate can not only provide energy for enterocyte regeneration but also modulate the intestine microbial community and contribute to the host’s health (Jiminez et al., 2017). Some of the studies reported that butyrate has an important role in the proper functioning of the immune system, nervous system, and energy metabolism (Koh et al., 2016; Fu et al., 2019). It was observed in the previous study that intestinal disease like ulcerative colitis is highly related to inadequate use of butyrate (Leonel and Alvarez-Leite, 2012). Butyrate could enhance epithelial barrier function, promote goblet cells mucus secretion, and reduce intestinal inflammation by reducing pro-inflammatory cytokines’ levels (Hamer et al., 2008; Gaudier et al., 2009; Guilloteau et al., 2010).

In our previous study, we found a significant decrease in concentrations of SCFAs, especially acetic acid and butyric acid in yaks (Li et al., 2022). We hypothesized that sodium acetate/sodium butyrate supplementation could alleviate diarrhea in animals, similar to how *Lactobacillus plantarum* alleviated diarrhea in a previous study by balancing gut microbiota and regulating SCFAs (Yue et al., 2020). The widely known lipopolysaccharide is an important membrane component of gram-negative bacteria, causing the inflammatory reaction, oxidative damage, and gut dysbiosis in hosts (Xu et al., 2021). Previous studies found that ROS were an important second messenger of the atypical domain (NOD)-like receptor containing pyrin domain 3 inflammasomes, and Caspase-1 was activated by NLRP3 and then cause inflammation reaction (Dashdorj et al., 2013; Sho and Xu, 2019; He et al., 2022). Hence, we conducted this study to explore the alleviation effect and potential mechanism of sodium acetate/sodium butyrate supplementation on LPS-induced diarrhea in mice *via* NLRP3/Caspase-1 signaling.

## Materials and methods

### Experiment design

**FIGURE 1:**
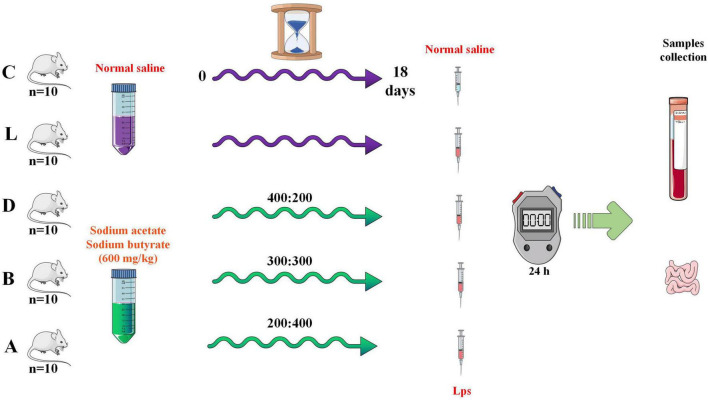
Study design for the experiment.

A total of 50, four weeks of age, ICR mice with an equal number of male and female animals (average weight of 18 ± 2 g) were purchased from Qing Long Shan Dong Wu Fan Zhi (Nanjing, China). After 30 days of rearing, mice were randomly divided into five groups, namely control (C), LPS (L), and treatment groups (A, B, and D). Group D (400: 200), B (300:300), and A (200: 400) were treated with 600 mg/kg sodium acetate/sodium butyrate solution *via* gavage for 18 days, while mice in C and L were treated with equal volume of normal saline. On day 19, mice in groups L, A, B, and D were treated with 20 mg/kg LPS (Solarbio life science, China), and after 24 hours mice were euthanized to collect serum, small intestine, and rectum samples (Figure 1). All animals were given normal water and feeds, and kept in the laboratory animal center of Nanjing Agricultural University. The body weights and diarrhea were documented.

### Hematoxylin and eosin staining

Duodenum, jejunum, and ileum samples from all the groups were preserved in 4% paraformaldehyde for at least 48 hrs and then processed for commercial H&E staining (Wuhan Pinuofei Biological Technology Co., Ltd., China). On an Olympus CX23 microscope with an integrated digital imaging analysis system, histological slices were examined (Olympus Co., Japan). The villus height and crypt depth were measured as depicted in the previous study (Xu et al., 2021).

### Antioxidative indices, cytokine levels, and NOs levels in serum

Blood samples of mice were centrifuged at 4,000 g for 10 min and stored at –20°C for future analysis. Antioxidant capacity was examined by detecting the levels of superoxide dismutase (SOD), glutathione peroxidase (GSH-px), total anti-oxidation capacity (T-AOC), and malondialdehyde (MDA) by utilizing commercial assay kits (Nanjing Jiancheng Bioengineering Institute, China). Meanwhile, the NO concentrations were determined *via* commercial assay kits (Nanjing Jiancheng Bioengineering Institute, China). The concentration of cytokines including interleukin 1 beta (IL-1β), interleukin 6 (IL-6), interleukin 10 (IL-10), and tumor necrosis factor-alpha (TNF-α) was measured by using commercial ELISA kits (Solarbio life science, China).

### Gut microbiota sequencing and analysis

The total microbial genomic DNA was extracted from the rectum contents of each mouse. The samples from groups C (*n* = 4), L (*n* = 4), and A (*n* = 4) were extracted utilizing the fast DNA Stool Mini Kit (Qiagen, German) according to the manufacturer’s specifications. The quantity and quality of all extracted DNA samples were examined by using NanoDrop 2000 UV-vis spectrophotometer (Thermo Scientific, USA) and agarose gel electrophoresis, respectively. Gene amplification of bacterial 16S rRNA gene was performed using the V3–V4 regions primers 338F (5′-ACTCCTACGGGAGGCAGCAG-3′) and 806R (5′-GGACTACHVGGGTWTCTAAT-3′). Then all amplicon products were purified by employing Vazyme VAHTSTM DNA Clean Beads (Vazyme, China) and quantified using the QuantiFluor™-ST (Promega, USA). At last, all samples were sequenced by using the Illumina MiSeq platform (Bioyi Biotechnology Co., Ltd. China) with MiSeq Reagent Kit v3.

All of the achieved sequencing raw data were cleaned using the DADA2 (Callahan et al., 2016) and Vsearch (Rognes et al., 2016) to generate accurate and reliable results for microbiome bioinformatic analysis through QIIME2 (2019.4)^1^ (Bokulich et al., 2018). Phylogenetic trees were constructed *via* mafft (Katoh et al., 2002) and FastTree (Price et al., 2010). Alpha-diversity metrics of Chao1 (Chao, 1984), observed species, Shannon (Shannon, 1948), Simpson (Simpson, 1997), Faith’s PD (Faith, 1992), Pielou’s evenness (Pielou, 1966), and Good’s coverage (Good, 1953) were estimated among samples. Beta diversity metrics of principal coordinate analysis (PCoA) (Alban, 2010), non-metric multidimensional scaling (NMDS) (Legendre and Montréal, 2003), and unweighted pair-group method with arithmetic means (UPGMA) were estimated among samples. Taxonomy was assigned to non-singleton amplicon sequence variants (ASVs) using the classify-sklearn naïve Bayes taxonomy classifier in the feature-classifier plugin (Bokulich et al., 2018) against the SILVA Release 132 Database^2^ (Quast et al., 2012). Tree diagram of classification levels and GraPhlAn evolutionary was generated *via* ggtree^3^. Krona species composition map was generated *via* KronaTools (v2.7)^4^ (Ondov et al., 2011). Significant difference analyses among different mouse groups were performed *via* PERMANOVA and Adonis in QIIME2 (2019.4). Venn^5^, heatmap, metagenomeSeq, LEFSe (Mahadevan et al., 2008; Segata et al., 2011), OPLS-DA (Mahadevan et al., 2008), and random forest analysis were carried to explore the significant difference in species. Network analysis (Faust and Raes, 2012) was performed to find potential keystone. The functional potential prediction was carried through the phylogenetic investigation of communities by the reconstruction of unobserved states (PICRUSt2) (Gavin et al., 2019) using MetaCyc^6^ and KEGG^7^ databases.

### RNA extraction and RT-qPCR analysis

Intestinal tissue RNA extraction from all mouse groups was performed by utilizing TRIzol reagent (Life Technologies, USA). All of the RNA samples were examined *via* denaturing formaldehyde gel electrophoresis and NanoDrop 2000 analyzer (Thermo Fisher Scientific, China) to validate their integrity and concentrations, respectively. Then commercial SuperScript™IV first strand cDNA synthesis kits (Invitrogen™, Thermo Fisher Scientific, USA) were used for translating RNA samples into cDNA under the guidance of the manufacturer’s specifications. Finally, qRT-PCR for all groups was carried out by using 25 uL of reaction mixtures consisting of 2 uL of intestinal tissues cDNA, 12.5 uL of Hieff UNICON^®^ Universal Blue qPCR SYBR Green Master Mix (Yeasen, China), 2uL of primers, and 8.5 uL nuclease-free water, then the procedure was performed in the StepOnePlus™ Real-Time PCR System (Applied Biosystems, USA). All sample reactions were repeated three times and the method of 2^–ΔΔCT^ was utilized for calculating gene relative quantification. All primer pairs used in the present study were synthesized by Sangon Biotech (China) and are shown in Table 1.

**TABLE 1:** Primers used in the present study.

Genes	Primer sequence (5′–3′)	Product size (bp)	Tm (°C)
ZO-1	F: CTGGTGAAGTCTCGGAAAAATG R: CATCTCTTGCTGCCAAACTATC	97	54
Occludin	F: TGCTTCATCGCTTCCTTAGTAA R: GGGTTCACTCCCATTATGTACA	155	54
Claudin	F: AGATACAGTGCAAAGTCTTCGA R: CAGGATGCCAATTACCATCAAG	86	54
Caspase-1	F: TGCCCTCATTATCTGCAACA R: GATCCTCCAGCAGCAACTTC	95	56
NLRP3	F: CATCAATGCTGCTTCGACAT R: TCAGTCCCACACACAGCAAT	118	56
β-actin	F: CTACCTCATGAAGATCCTGACC R: CACAGCTTCTCTTTGATGTCAC	90	54

### Statistical analysis

All the generated data were evaluated *via* ANOVA, Student’s t-test, Kruskal–Wallis, and Dunn’s test *via* IBM SPSS (22.0) software. Data presented as means ± SD and statistically significant are considered when *P* < 0.05.

## Results

### Effects of sodium acetate/sodium butyrate supplementation on body weights and intestinal damage induced by LPS

**FIGURE 2:**
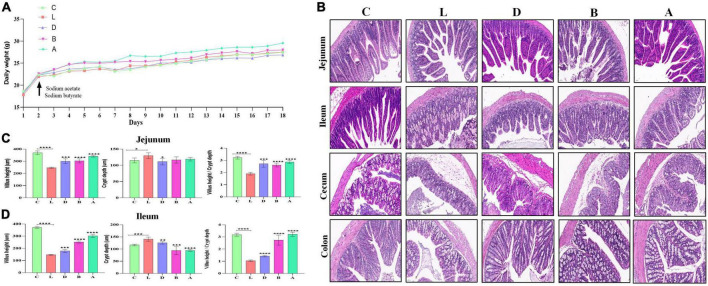
Effects of sodium acetate/sodium butyrate supplementation on body weight, diarrhea score, and intestinal damage induced by LPS. **(A)** Mouse daily weights, **(B)** H&E staining analysis of small intestine of mouse, **(C)** villus height, crypt depth, and villus height/crypt depth ratio of Jejunum, and **(D)** villus height, crypt depth, and villus height/crypt depth ratio of Ileum. Scale bar 50 μm. Significance is presented as *p < 0.05, **p < 0.01, ***p < 0.001, and ****p < 0.0001; data are presented as the mean ± SEM (*n* = 3).

The mice were weighed on a daily basis and the weight of the mice in group A was slightly higher than mice in other groups (Figure 2A). The diarrhea was found in the mouse of group L (induced by LPS), whereas sodium acetate/sodium butyrate supplementation alleviated diarrhea, especially in mice of group A. The intestines’ morphology was examined *via* H&E staining and found that LPS caused a decrease in the villus height (*p* < 0.0001), an increase in the crypt depth (*p* < 0.05), and it also lowered the villus height to crypt depth ratio (*p* < 0.0001). Whereas, sodium acetate/sodium butyrate supplementation resulted in a significant increase in the villus height (*p* < 0.001), decrease in the crypt depth (*p* < 0.01), and increase in the villus height to crypt depth ratio (*p* < 0.001), especially in the mice of groups B and A (Figure 2).

### Effects of sodium acetate/sodium butyrate on the inflammation response and oxidative stress of mouse induced by LPS

**FIGURE 3:**
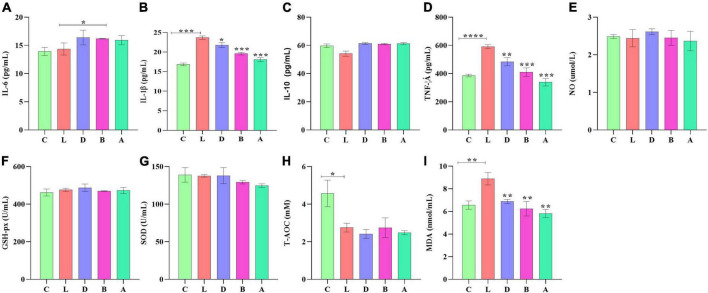
Sodium acetate/sodium butyrate supplementation improved the inflammation response and oxidative stress of mouse induced by LPS. The concentrations of inflammatory cytokines IL-6 **(A)**, IL-1β **(B)**, IL-10 **(C)**, TNF-α **(D)**, and NO **(E)** in serums. Oxidative status indices levels of GSH-px **(F)**, SOD **(G)**, T-AOC **(H)**, and MDA **(I)** in serums. Significance is presented as *p < 0.05, **p < 0.01, ***p < 0.001, and ****p < 0.0001; data are presented as the mean ± SEM (*n* = 3).

In mice serum, no obvious difference was found in IL-6, IL-10, NO, GSH-px, and SOD levels among the control group and LPS-induced groups, respectively. T-AOC in group L was significantly low than in group C (*p* < 0.05), while there was no marked difference between group L and treated groups D, B, and A, respectively. In group C, the serum level of mice induced by LPS was found prominently high for IL-1β (*p* < 0.001), TNF-α (*p* < 0.001), and MDA (*p* < 0.01), respectively. However, in the serum of groups D, B, and A, sodium acetate/sodium butyrate supplementation caused a remarkable decrease in IL-1β (*p* < 0.001), TNF-α (*p* < 0.01), and MDA (*p* < 0.01), respectively (Figure 3).

### Effects of sodium acetate/sodium butyrate supplementation on the structure and diversity of mouse gut microbiota

**FIGURE 4:**
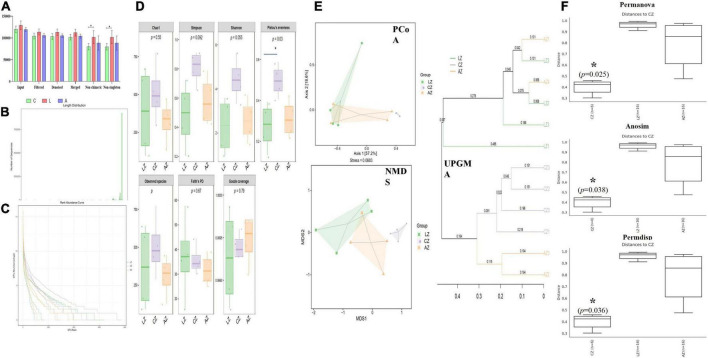
Sodium acetate/sodium butyrate supplementation restored the structure and diversity of mouse gut microbiota affected by LPS. **(A)** Sequencing data statistical analysis, **(B)** length distribution of sequencing data, **(C)** rank abundance curve, **(D)** alpha-diversity index analysis, **(E)** beta diversity analysis, and **(F)** group difference analysis. Significance is presented as *p < 0.05, **p < 0.01, ***p < 0.001, and ****p < 0.0001; data are presented as the mean ± SEM (*n* = 4).

In the current study, over 110 000, 95 000, 90 000, 94 000, 69 000, and 69 000 of input, filtered, denoised, merged, non-chimeric, and non-singleton data were achieved, respectively, in different mouse samples (Table 2). A significant difference in non-chimeric (*p* < 0.05) and non-singleton (*p* < 0.05) was found between groups C and L (Figure 4A). The majority of sequence lengths were around 430 bp (Figure 4B). As shown in Figure 4C, flatness broken lines were present in all samples, which reflected the evenness of OTUs composition in samples. Alpha-diversity index of Chao1, Simpson, Shannon, Pielou’s evenness, observed species, Faith’s PD, and Goods coverage is shown in Table 3. There was a significant difference in only Pielou’s evenness between groups C and A (*p* < 0.05) (Figure 4D). Beta diversity analysis indicated a far distance of points in samples of group L, whereas relatively near points were found in group A *via* PCoA and NMDS analyses, respectively (Figure 4E). UPGMA analysis found that the branch length in group C was relatively shorter in group A compared with samples in group L (Figure 4E). Significant differences between groups were found in groups A and C through PERMANOVA (*p* < 0.05), ANOSIM (*p* < 0.05), and PERMDISP (*p* < 0.05), respectively (Figure 4F).

**TABLE 2:** Statistical analysis of sample sequencing data.

Sample ID	Input	Filtered	Denoised	Merged	Non-chimeric	Non-singleton
CZ1	117228	103506	102080	98791	70298	69958
CZ2	110721	95414	94891	94063	87694	87636
CZ3	127184	110284	109548	107874	81870	81757
CZ4	124309	108080	107080	105393	79170	79009
LZ1	138930	118886	118448	118127	112023	111994
LZ2	136031	120329	120002	119787	117181	117170
LZ3	119805	107500	106496	104659	87609	87411
LZ4	120971	108244	107518	106137	90221	90048
AZ1	114290	102104	101618	100822	90342	90275
AZ2	117460	102209	101350	100174	85482	85389
AZ3	123722	110608	110272	110016	108806	108790
AZ4	121781	107460	106791	105671	69110	69002

**TABLE 3:** Statistical analysis of alpha-diversity index.

Sample	Chao1	Simpson	Shannon	Pielou’s evenness	Observed species	Faith’s PD	Goods coverage
CZ1	776.424	0.910572	5.64342	0.588308	772.1	44.4865	0.999433
CZ2	301.297	0.655669	3.32679	0.406681	290.1	30.4710	0.999602
CZ3	456.026	0.881407	4.67368	0.53205	440.9	33.3046	0.999271
CZ4	525.863	0.782378	4.13132	0.458692	514.4	35.1551	0.999288
LZ1	182.193	0.204933	0.978133	0.132322	168.0	57.6407	0.999628
LZ2	97.6659	0.398078	1.464920	0.22345	94.1	15.9611	0.999873
LZ3	672.409	0.600411	3.21118	0.344443	640.4	38.3927	0.998668
LZ4	600.000	0.722918	3.59158	0.392133	571.7	35.7839	0.998896
AZ1	309.775	0.457996	2.07760	0.253912	290.5	25.4681	0.999414
AZ2	472.144	0.788589	3.72562	0.422906	448.7	34.7309	0.999080
AZ3	139.305	0.426415	1.52562	0.217816	128.4	39.1726	0.999709
AZ4	372.352	0.6599	3.01108	0.352894	370.3	27.8166	0.999680

### Effects of sodium acetate/sodium butyrate solution supplementation on the taxon composition of mouse gut microbiota

**FIGURE 5:**
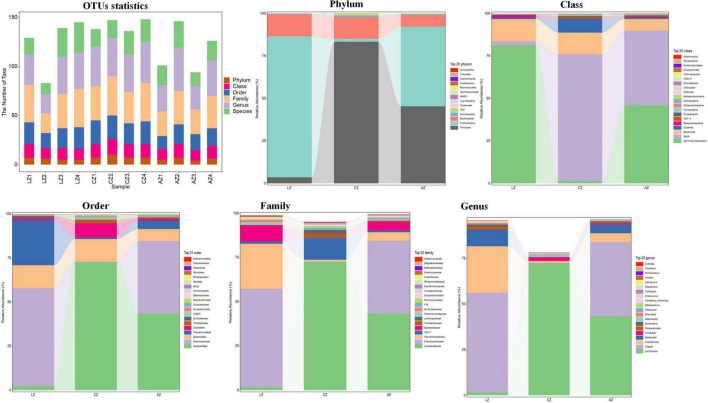
Effect of dietary sodium acetate/sodium butyrate supplementation on the relative abundance of gut microbiota in different taxa levels.

The number of taxa contained in different levels of phylum, class, order, family, genus, and species is shown in Figure 5. At the phylum level, the dominating phyla in group L were *Proteobacteria* (83.02%) and *Bacteroidetes* (13.00%). While *Firmicutes* (83.36%), *Bacteroidetes* (12.80%), *Firmicutes* (45.35%), and *Proteobacteria* (46.88%) were the main phyla in groups C and A. At the Class level, the main classes were *Gammaproteobacteria* (81.28%) and *Bacteroidia* (12.99%) in group L, while *Bacilli* (74.72%), *Bacteroidia* (12.80%), Clostridia (8.08%), *Gammaproteobacteria* (45.90%), *Bacilli* (44.04%), and *Bacteroidia* (6.80%) were the dominant classes in groups C and A, respectively. At the order level, the primary classes were *Enterobacteriales* (55.60%), *Pseudomonadales* (25.65%), and *Bacteroidales* (12.99%), while *Lactobacillales* (72.68%), *Bacteroidales* (12.80%), Clostridiales (8.08%), *Lactobacillales* (43.52%), *Enterobacteriales* (41.07%), and *Bacteroidales* (6.80%) were the staple orders in groups C and A, respectively. At the family level, the dominating families in group L were *Enterobacteriaceae* (55.59%), *Pseudomonadaceae* (25.47%), and *Bacteroidaceae* (9.09%), while the main families were *Lactobacillales* (72.47%) and *S24-7* (12.50%) in group C, whereas *Lactobacillales* (43.35), *Enterobacteriaceae* (41.07%), and *Bacteroidaceae* (4.84%) in group A, respectively. At the genus level, the major genera in group L were *Shigella* (54.62%), *Pseudomonas* (25.42%), and *Bacteroides* (9.09%), while *Lactobacillus* (72.45%), *unidentified_S24-7* (12.50), *Lactobacillus* (43.34%), *Shigella* (40.71), and *Bacteroides* (4.84%) were the dominating genera in groups C and A (Figure 5).

**FIGURE 6:**
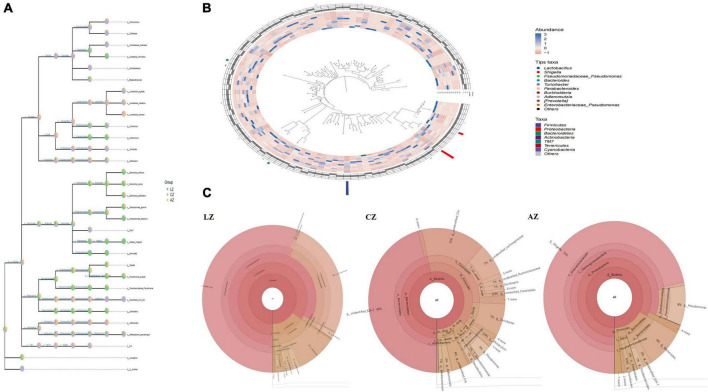
Species composition analysis of mouse gut microbiota. **(A)** Classification levels tree diagram, **(B)** GraPhlAn evolutionary tree diagram, and **(C)** Krona species composition diagram.

The classification levels tree diagram showed that larger sectors of *Ruminococcus*, *Oscillospira*, *Lactobacillus vaginalis*, *Lactobacillus helveticus*, *Lactobacillus hamsteri*, *Turicibacter*, *Allobaculum*, *Adlercreutzia*, and *F16* with light orange and lavender color in groups A and C, respectively, while larger sectors of *Candidatus Arthromitus, Lactococcus, Enterococcus, Bacteroides uniformis, Bacteroides caccae, Bacteroides acidifaciens, Parabacteroides gordonii, Parabacteroides distasonis, Alistipes finegoldii, Prevotella, Shigella, Pseudomonas syringae*, and *Pseudomonas Pseudomonas* with green color were found in group L (Figure 6A). GraPhlAn evolutionary tree diagram showed that the abundance of *Lactobacillus*, *Shigella*, *Pseudomonas Pseudomonas, Bacteroides, Turicibacter, Parabacteroides, Burkholderia, Adlercreutzia, Prevotella*, and *Enterobacteriaceae Pseudomonas* depicted with various colors were found significantly different among different mouse groups (Figure 6B). Krona species composition diagram indicated that the main genera were *Shigella* (72%), *Bacteroides* (9%), and *Pseudomonas* (8%) in group L, while *Shigella* (56%), *Pseudomonas* (26%), and *Bacteroides* (9%) in group A, and unidentified *S34-7* (45%), unidentified *Clostridia* (15%), *Lachnospiraceae* (7%), and *Turicibacter* (7%) in group C, respectively (Figure 6C).

**FIGURE 7:**
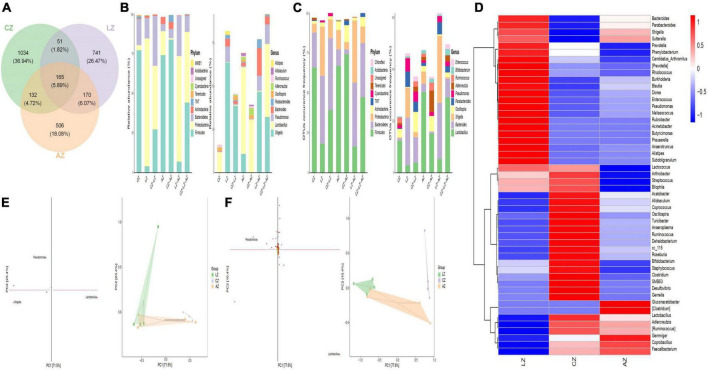
Different species and marker species analysis in mouse microbiota induced by LPS. **(A)** Venn diagram, **(B)** bar chart of ASV/OTU numbers in different regions of Venn diagram, **(C)** bar graphs of ASV/OTU abundance in different regions of the Venn diagram, **(D)** genera composition heatmap, **(E)** PCA, and **(F)** OPLS-DA.

To find different species and markers, species in mouse microbiota induced by LPS, we performed the Venn diagram, bar chart of ASV/OTU numbers in different regions of the Venn diagram, bar graphs of ASV/OTU abundance in different regions of the Venn diagram, Genera composition heatmap, PCA, and OPLS-DA analysis. Results showed that 216 (7.71%) OTUs were shared in groups C and L, while 335 (11.96%) OTUs were shared in groups C and A (Figure 7A). Then ASV/OTU abundance was explored in different regions of the Venn diagram. The results showed that at the phylum level, groups C and A shared *Firmicutes, Proteobacteria, Bacteroidetes, Actinobacteria*, and *TM7* Phyla, while groups C and L shared *Firmicutes, Proteobacteria*, and *Bacteroidetes* Phyla. At the genus level, groups C and L shared *Shigella*, *Lactobacillus, Pseudomonas, Bacteroides, Parabacteroides, Oscillospira, Allobaculum*, and *Ruminococcus* Genera, while groups C and A shared *Lactobacillus, Adlercreutzia, Oscillospira, Ruminococcus*, and *Allobaculum* genera (Figure 7B). ASV/OTU number analysis found that at the Phylum level, groups C and L shared *Firmicutes, Bacteroidetes, Proteobacteria, Actinobacteria*, and *Cyanobacteria*, while groups C and A shared *Firmicutes, Bacteroidetes, Proteobacteria, Actinobacteria, TM7*, and *Tenericutes* Phyla. At the genus level, groups C and L shared *Lactobacillus, Bacteroides, Shigella, Oscillospira, Parabacteroides, Pseudomonas, Adlercreutzia, Ruminococcus*, and *Enterococcus*, while groups C and A shared *Lactobacillus, Bacteroides, Oscillospira, Pseudomonas, Adlercreutzia, Ruminococcus, Bifidobacterium*, and *Enterococcus* genera (Figure 7C). It is depicted in the heatmap that the abundance of *Bacteroides, Parabacteroides, Shigella, Prevotella, Phenylobacterium, Candidatus Arthromitus, Prevotella, Rhodococcus, Burkholderia, Blautia, Dorea, Enterococcus, Pseudomonas, Melissococcus, Rubrobacter, Acinetobacter, Butyricimonas, Prauserella, Anaerotruncus, Alistipes*, and *Subdoligranulum* shown in red color in group L were obviously higher than in groups C and A, while *Lactobacillus, Adlercreutzia, Ruminococcus, Coprobacillus*, and *Faecalibacterium* shown in blue color in group L were significantly lower than in groups C and A (Figure 7D). PCA analysis represents that the main genera among mouse groups were *Lactobacillus, Shigella*, and *Pseudomonas*. The distance between points of group A (green) projected on the coordinate axis was clearly farther than groups A (light orange) and C (lavender), which revealed a difference between group L, and groups A and C, respectively (Figure 7E). Also, OPLS-DA analysis revealed similar results to PCA analysis (Figure 7F).

**FIGURE 8:**
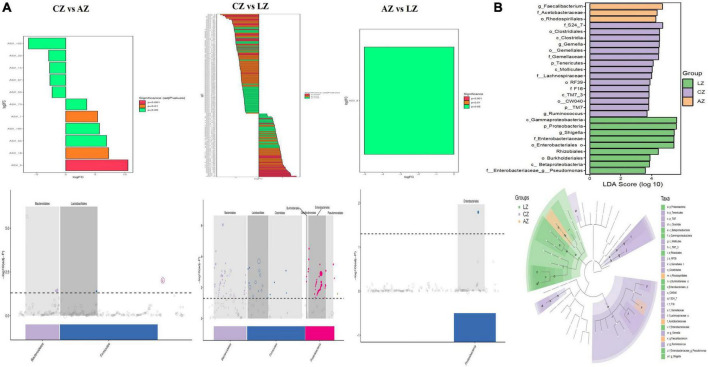
Gut microbiota difference analysis between mouse groups. **(A)** MetagenomeSeq analysis and **(B)** LEfSe analysis.

For further investigation, MetagenomeSeq analysis was performed and different genera like *Bacteroidales* and *Lactobacillales* were found significantly on the upside of the broken lines with decreased ASV 102 (*p* < 0.05), ASV 23 (*p* < 0.05), ASV 14 (*p* < 0.05), and ASV 53 (*p* < 0.05), increased ASV 73 (*p* < 0.05), ASV 1 (*p* < 0.01), ASV 194 (*p* < 0.05), ASV 50 (*p* < 0.05), ASV 18 (*p* < 0.01), and ASV 5 (*p* < 0.001) between groups C and A. Different genera like *Bacteroidales*, *Lactobacillales*, *Clostridiales*, *Burkholderiales*, *Desulfovibrionales*, *Enterobacteriales*, and *Pseudomonadales* were found significantly in groups C and L, with 107 decreased ASV and 86 increased ASV (Figure 8A). Biomarker bacteria in mouse groups were uncovered by using LEfSe analysis, which were from the class *Gammaproteobacteria*, phylum *Proteobacteria*, genus *Shigella*, family *Enterobacteriaceae*, order *Enterobacteriales, Rhizobiales*, order *Burkholderiales*, class *Betaproteobacteria*, and genus *Pseudomonas* in group L (green color), family *S24-7*, order *Clostridiales*, class *Clostridia*, genus *Gemella*, order *Gemellales*, family *Gemellaceae*, phylum *Tenericutes*, class *Mollicutes*, family *Lachnospiraceae*, order *RF39*, family *F16*, class *TMT-3*, order *CW040*, phylum *TM7*, and genus *Ruminococcus* in group C (lavender), genus *Faecalibacterium*, family *Acetobacteraceae*, and order *Rhodospirillales* in group A (light orange) (Figure 8B).

**FIGURE 9:**
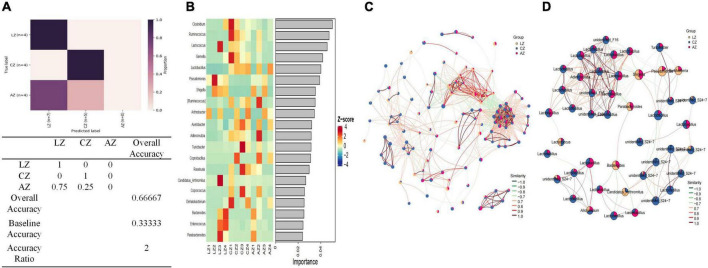
Random Forests and network analysis. **(A)** Model accuracy, **(B)** ASV taxa heatmap, **(C)** network analysis, and **(D)** subnetwork analysis of dominant genera.

Random forest analysis was performed with an accuracy ratio of 2 (Figure 9A). Important genera among mouse groups are shown in Figure 9B including *Clostridium*, *Ruminococcus*, *Lactococcus*, *Gemella*, etc. Network analysis revealed that there were more edges between groups A and C than between groups L and C, which inferred a higher similarity between groups A and C (Figure 9C). The dominant genera in the network were *unidentified F16*, *Lactobacillus*, *Adlercreutzia*, *unidentified S24-7*, and *Turicibacter* between groups A and C, while *Lactococcus* and *Allobaculum* were the main genera in the network between groups L and C (Figure 9D).

**FIGURE 10:**
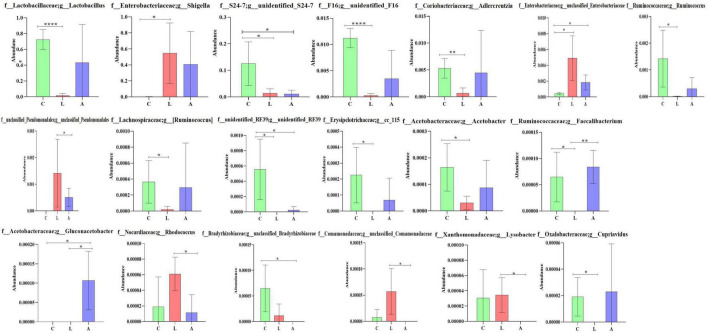
Comparing genera difference of mouse microbiota. Significance is presented as *p < 0.05, ^**^p < 0.01, ^***^p < 0.001, and ^****^p < 0.0001; data are presented as the mean ± SEM (*n* = 4).

By comparing the abundance of genera of mouse microbiota, 19 genera were detected among mouse groups. The abundance of *Lactobacillus* (*p* < 0.0001), *unidentified F16* (*p* < 0.0001), *Adlercreutzia* (*p* < 0.01), *Ruminococcu*s (*p* < 0.05), [*Ruminococcus*] (*p* < 0.05), *Acetobacter* (*p* < 0.05), *cc 115* (*p* < 0.05), and *Cupriavidus* (*p* < 0.05) in group L was obviously lower than group C, respectively. While *Shigella* (*p* < 0.05) was prominently higher in group L than in group C. *Unidentified_S24-7* and *unclassified RF39* were significantly higher in group C than in groups A (*p* < 0.05) and L (*p* < 0.05), respectively, while *unclassified Enterobacteriaceae* was significantly lower in group C than in groups A (*p* < 0.05) and L (*p* < 0.05), respectively. The abundance of unclassified *Pseudomonadales* (*p* < 0.05), *Rhodococcus* (*p* < 0.05), unclassified *Comamonadaceae* (*p* < 0.05), and *Lysobacter* (*p* < 0.05) in group L was conspicuously higher than group A, respectively. The abundance of *Faecalibacterium* in group L was clearly lower than groups A (*p* < 0.05) and L (*p* < 0.01), respectively. The abundance of *Gluconacetobacter* in group A was prominently higher than groups L (*p* < 0.05) and C (*p* < 0.01), respectively. The abundance of *unclassified Bradyrhizobiaceae* in group C was clearly higher than in group A (*p* < 0.05) (Figure 10).

In summary, different analysis methods include relative abundance taxa, classification levels tree diagram, GraPhlAn evolutionary tree diagram, Krona species composition diagram, Venn Diagram, Genera composition heatmap, PCA, OPLS-DA, metagenomeSeq analysis, LEfSe analysis, and random forests and network analyses demonstrated that LPS challenge changed microbiota composing in mice; however, sodium acetate/sodium butyrate supplementation could partly restore the gut microbiota in animals.

### Effects of sodium acetate/sodium butyrate solution supplementation on the function of mouse gut microbiota

**FIGURE 11:**
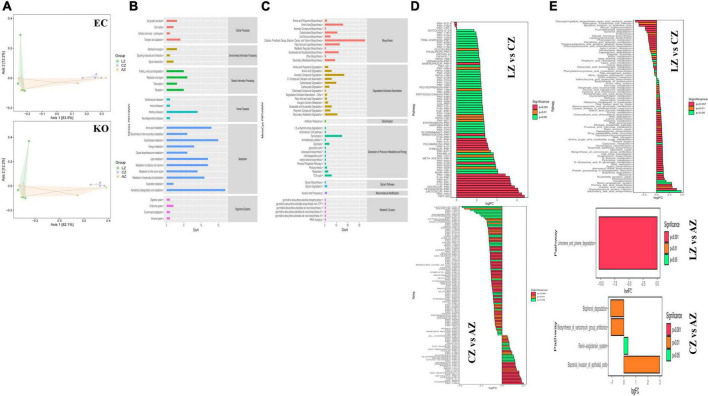
Potential function prediction analysis of mouse microbiota. **(A)** PCoA, **(B)** KEGG, **(C)** MetaCyc, **(D)** MetaCyc metabolic pathways, and **(E)** KEGG metabolic pathways.

PICRUSt2 was utilized for potential function prediction analysis of mouse microbiota. PCoA analysis found that closer points are projected on the coordinate axis in groups A and L in both functional units of KO and EC (Figure 11A), which revealed a more similar functional composition in these groups. KEGG analysis showed that the main pathways were related to metabolism (Figure 11B). MetaCyc analysis showed that the main pathways were related to biosynthesis, degradation/utilization/assimilation, and generation of precursor metabolite and energy (Figure 11C). MetaCyc metabolic pathways comparing analysis found 44 (*p* < 0.05), 9 (*p* < 0.01), and 20 (*p* < 0.001) significant different pathways between groups C and L, 37 (*p* < 0.05), 27 (*p* < 0.01), and 53 (*p* < 0.001) significant different pathways between groups A and C, respectively (Figure 11D). KEGG metabolic pathways comparing analysis revealed that 15 (*p* < 0.05), 15 (*p* < 0.01), and 51 (*p* < 0.001) significant different pathways between groups C and L, while one (*p* < 0.001) significant different pathway between groups A and C was examined (Figure 11E).

### Expression of ZO-1, Occludin, Claudin, Caspase-1, and NLRP3 genes in mouse intestines

Relative gene expression of Zonula occludens 1 (ZO-1), Occludin, Claudin, Caspase-1, and NLRP3 were detected by employing qRT-PCR. LPS induction prominently upregulated the expression of ZO-1 (*p* < 0.01) and NLRP3 (*p* < 0.0001) in mice of group L; however, sodium acetate/sodium butyrate solution supplementation downregulated the expression of ZO-1 (*p* < 0.05) and NLRP3 (*p* < 0.05) genes in treated mice. Furthermore, change in LPS clearly downregulated the expression of Occludin (*p* < 0.001), Claudin (*p* < 0.0001), and Caspase-1 (*p* < 0.0001) genes in group L, while sodium acetate/sodium butyrate solution supplementation upregulated those gene expressions in treated groups of D, B, and A (Figure 11).

### The correlation among differential bacteria, detection indices, and gene expressions

**FIGURE 12:**
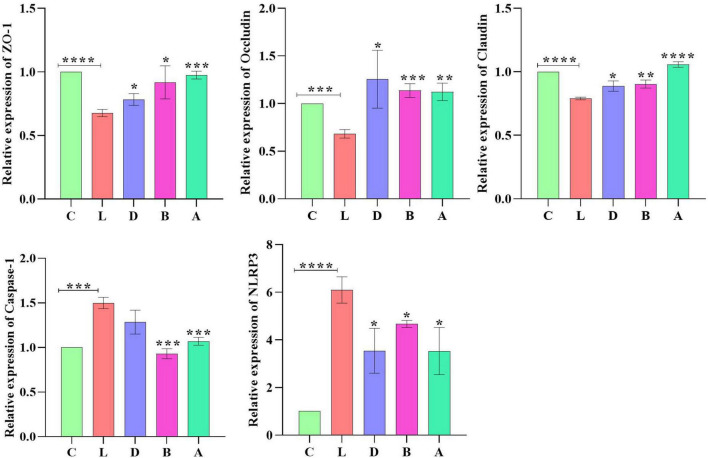
Relative expression analysis of ZO-1, Occludin, Claudin, Caspase-1, and NLRP3 *via* qRT-PCR. Significance is presented as *p < 0.05, **p < 0.01, ***p < 0.001, and ****p < 0.0001; data are presented as the mean ± SEM (*n* = 3).

Correlation assessment between differential bacteria (abundance of top 20 genera), intestine morphology indices, inflammatory cytokines, oxidative indices, and gene expressions was performed through Statistical Analysis System. Results showed that *Lactobacillus*, *Turicibacter*, *Roseburia*, *Allobaculum*, *Bifidobacterium*, *Faecalibacterium*, *Ruminococcus*, *Coprococcus*, *cc_15*, *Gemella*, *Pediococcus*, *Butyricicoccus*, and *Cupriavidus* were positively related to villus height and villus height to crypt depth ratio, while *Rhodococcus*, *Phenylobacterium*, *Lysobacter*, and *W22* were positively related to villus height. *Lactobacillus*, *Allobaculum*, *Rhodococcus*, *Butyricicoccus*, and *Ralstonia* were found positively related to antioxidant ability. *Pseudomonas*, *Faecalibacterium*, *Faecalibacterium*, *Candidatus Arthromitus*, *Ruminococcus*, *Roseburia*, *Gemella*, *Faecalibacterium*, *Gluconacetobacter*, *Rhodococcus*, *Anaeroplasma*, (*Clostridium*), *Phenylobacterium*, and *Aminobacter* were positively related to inflammatory cytokines. *Pseudomonas*, *Faecalibacterium*, *Gluconacetobacter*, *Rhodococcus*, (*Clostridium*), and *Gluconobacter* were positively related to tight junction proteins, while *Adlercreutzia*, *Candidatus Arthromitus*, *Lactococcus*, *Ruminococcus*, *Coprococcus*, *Acinetobacte*r, *cc_115*, *Gemella*, *Anaeroplasma*, *Phenylobacterium*, *Lysobacter*, and *W22* were negatively related to tight junction proteins. Adlercreutzia, Ruminococcus, Coprococcus, cc_115, Roseburia, Gemella, and Anaeroplasma were negatively related to expressions of Caspase-1 and NLRP3 (Figure 12).

## Discussion

Though various kinds of measures have been implemented so far to fight against diarrhea, there is still a long way to go (Cho et al., 2013). A recent review found that higher antimicrobial resistance was associated with the drugs being used for the treatment of diarrhea (Zhang H. et al., 2022; Zhang X. et al., 2022); therefore, establishing a method to positively regulate the gut microbiota to ameliorate diarrhea is of great importance and meaningful (Coelho et al., 2022).

In the present study, mice were supplemented with sodium acetate/sodium butyrate before inducing diarrhea by utilizing LPS. Serve damage to intestines particularly jejunum, ileum, cecum, and colon was examined (Figure 1B), which was in accordance with previous results (He et al., 2022). An obvious decrease in villus height, increase in crypt depth, and lowered villus height to crypt depth ratio were uncovered in group C, while interestingly, sodium acetate/sodium butyrate supplementation could significantly increase the villus height, decrease the crypt depth, and increase the villus height to crypt depth ratio in both jejunum and ileum, especially in groups B and A (Figures 1C,D). The current results demonstrated that sodium acetate/sodium butyrate could improve intestinal integrity deteriorated by LPS, as intestinal barrier integrity is considered an important indicator of intestinal health (Liu H. et al., 2022). The current results may reveal that sodium acetate/sodium butyrate supplementation is a hopeful and effective method to alleviate diarrhea in animals.

To explore the potential mechanisms, we detected the gene expressions of tight junction proteins in intestines. Among them Occludin and Claudins were recognized as important components of intestinal permeability (Chen et al., 2015). The expression of Occludin in group L was significantly lower than in group C, while expression levels of Occludin increased in sodium acetate/sodium butyrate-treated groups (Figure 11). The current results were in line with a study on inflammatory bowel disease in humans with the downregulation of Occludin (Chen et al., 2015). The expression of Claudin in group L was significantly lower, which is in line with previous results (He et al., 2022); however, with the treatment of sodium acetate/sodium butyrate, the expression of Claudin upregulated significantly. The plaque protein ZO1 is an adaptor connecting trans-membrane protein with the peri-junctional actomyosin ring (Ulluwishewa et al., 2011). Like previous results found lower expressions of ZO-1 (Cao et al., 2022; He et al., 2022), the expression of ZO-1 in group L was significantly higher than in group C, while expression levels of ZO-1 decreased in sodium acetate/sodium butyrate-treated groups. The current findings suggested that acetate/sodium butyrate could improve intestinal barrier function by modulating tight junction gene expressions.

Reactive oxygen species are widely known for their important role in inflammatory diseases like colitis. The intestinal tissue injury is mediated through the administration of the antioxidants (Dashdorj et al., 2013; Aziz et al., 2021; Hassan et al., 2021; Liu et al., 2021; Murtaza et al., 2021). Oxidative agents of SOD, T-AOC, GSH-Px, and MDA are commonly known important enzymes related to oxidative stress, which may cause intestinal damage (Mehmood et al., 2019; Liu J. et al., 2022). In mice serums, no obvious difference was found in GSH-px and SOD between the control group and LPS-induced groups, respectively, while a prominently higher level of MDA (*p* < 0.01) was detected in group L; however, there was a significant decrease in MDA (*p* < 0.01) levels in sodium acetate/sodium butyrate supplemented groups D, B, and A, respectively (Figure 3), suggesting that sodium acetate/sodium butyrate improves intestinal oxidative damage by reducing MDA contents. Previous studies found that the activation of Caspase-1 by NLRP3 inflammasome could cause inflammation reaction by promoting the maturation of IL-1β, and ROS was a generally accepted second key messenger of NLRP3 inflammasome (Dashdorj et al., 2013; Sho and Xu, 2019; He et al., 2022). LPS inducing obviously upregulated the expression of NLRP3 (*p* < 0.0001) in mice in group L; however, sodium acetate/sodium butyrate solution supplementation downregulated the expression of NLRP3 (*p* < 0.05) genes in treated mice. Also, the LPS challenge clearly upregulated the Caspase-1 (*p* < 0.0001) gene in group L, while sodium acetate/sodium butyrate solution supplementation downregulated it in treated groups D, B, and A (Figure 11). TNF-α is an important cytokine in inflammation (Zelová and Hošek, 2013), Significantly higher IL-1β and TNF-α (*p* < 0.001) were found in group C, which was consistent with previous results found higher inflammatory factors and upregulation of NLRP3 in HUVEC cells (Liu et al., 2021). However, sodium acetate/sodium butyrate supplementation significantly decreased IL-1β and TNF-α in groups D, B, and A, respectively (Figure 3), demonstrating that sodium acetate/sodium butyrate could reduce intestine inflammation caused by LPS by alleviating oxidative damage *via* the downregulation of NLRP3 and Caspase-1.

**FIGURE 13:**
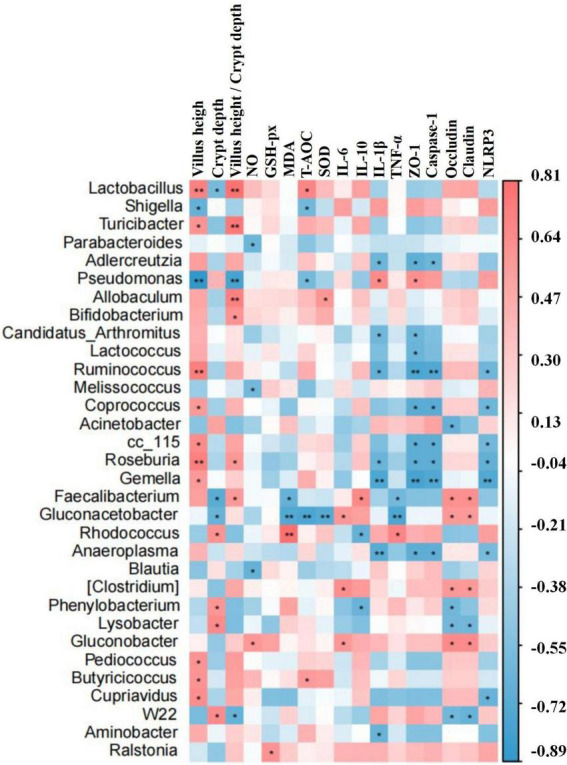
The differential bacteria of mouse were notably corrected with the indices of inflammatory cytokines, oxidative status, intestine morphology, and gene expressions by the statistical analysis system. Significance is presented as *p < 0.05 and **p < 0.01; data are presented as the mean ± SEM (*n* = 4).

Previous study found that LPS-induced ROS caused gut microbiota dysbiosis in piglets (Xu et al., 2021). Similar results were found in the current results in mice. The structure and diversity of mouse gut microbiota were significantly altered in LPS-induced mice, as determined by alpha and beta diversity analysis (Figure 13), taxa analysis at different levels (Figure 5), Classification levels tree diagram, and GraPhlAn evolutionary tree diagram analysis (Figures 6A,C). Potential function prediction analysis by PICRUSt2 and KEGG analysis found that LPS significantly changed the main pathways of mice (Figure 11). In consistent with previous studies (Yue et al., 2020; Xu et al., 2021; Chang et al., 2022), sodium acetate/sodium butyrate supplementation restored the structure, diversity, and partly function of mouse gut microbiota. Venn diagram, heatmap, PCA, OPLS-DA (Figure 7), metagenomeSeq (Figure 8), and random forests and network analysis (Figure 9) analyses were performed to reveal different species and their markers in mice microbiota induced by LPS. A total of 19 genera were detected among mouse groups (Figure 10). LPS challenge decreased the abundance of *Lactobacillus, unidentified F16, unidentified_S24-7, Adlercreutzia, Ruminococcus, unclassified Pseudomonadales, (Ruminococcus), Acetobacter, cc 1, Rhodococcus, unclassified Comamonadaceae, Faecalibacterium*, and *Cupriavidus*, while increased *Shigella, Rhodococcus, unclassified Comamonadaceae*, and *unclassified Pseudomonadales* in group L. Interestingly sodium acetate/sodium butyrate supplementation increased *Lactobacillus, unidentified F16, Adlercreutzia, Ruminococcus, (Ruminococcus), unidentified F16, cc 115, Acetobacter, Faecalibacterium*, and *Cupriavidus*, while decreased *Shigella, unclassified Enterobacteriaceae, unclassified Pseudomonadales, Rhodococcus*, and *unclassified Comamonadaceae*. *Lactobacillus* genus bacteria are probiotic microorganisms that have beneficial effects on to host (Rajab et al., 2020), and previous studies demonstrated that *Lactobacillus* could improve diarrhea in infants and piglets (Hu et al., 2019; Wang et al., 2020). A previous study reported that the genus *Adlercreutzia* was related to body weight loss (Liu Z. et al., 2022), the abundance of *Ruminococcus* was significantly decreased in diarrhea piglets (Liu C. et al., 2019; Liu G. et al., 2019). The changes of Adlercreutzia and Ruminococcus in the current study were consistent with previous findings. Bacteria from the *Acetobacter* genus are related to lignin degradation and intestine metabolism (Wu et al., 2022), and the decreasing of this genus may affect the metabolism of animals. Also, these bacteria can generate acetate (Zhang H. et al., 2022), which confirmed that diarrhea was related to the inefficiency of acetate. Bacteria of *Rhodococcus* are from natural environments (Alvarez et al., 2021), which may contrite little to diarrhea. A previous study reported that *unclassified Comamonadaceae* were related to the degradation of organics (Zhang et al., 2020), which may infer that LPS inducing could affect organics metabolism. The lower abundance of *Faecalibacterium* was consistent with the previous study observed in patients with colitis and Crohn’s disease (Ponziani et al., 2020). *Faecalibacterium* could cause inflammation through decreased SCFAs (Nishiwaki et al., 2020), which showed that LPS-induced diarrhea may attribute to the absence of SCFAs. Bacteria from *Cupriavidus* genus are related to biodegradation and biodetoxifcation, which may indicate that LPS could reduce the degradation (Al-Nussairawi et al., 2020) and detoxification ability of mouse. *Shigella spp.* are well-known pathogens causing bacterial dysentery (Liu C. et al., 2019), which should reveal the reason of diarrhea caused by LPS. Bacteria from the top 20 abundance genera related to intestine damage, oxidation resistance, inflammatory factor, and gene expressions are shown in Figure 12, which demonstrated that sodium acetate/sodium butyrate could regulate microbiota to improve diarrhea induced by LPS in mice.

## Conclusion

**FIGURE 14:**
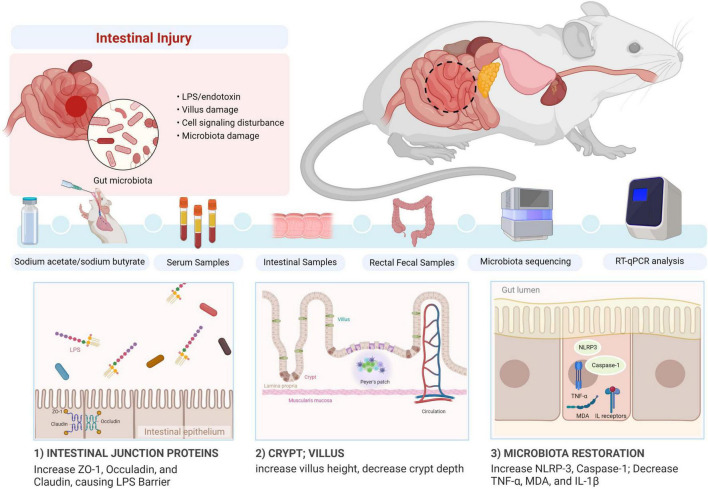
Summary of the potential mechanism of acetate/sodium butyrate alleviates LPS-induced diarrhea in mice.

In conclusion, we revealed that sodium acetate/sodium butyrate could alleviate LPS-induced diarrhea in mice by increasing beneficial bacteria and decreasing pathogens, which could regulate oxidative damage and inflammatory responses *via* NLRP3/Caspase-1 signaling (Figure 14). The current results may give insights into the prevention and treatment of diarrhea.

## Data availability statement

The data presented in this study are deposited in the https://www.ncbi.nlm.nih.gov/ repository, accession numbers: PRJNA872847 and SAMN30471364-SAMN30471375.

## Ethics statement

This animal study was reviewed and approved by Laboratory Animals Research Centre of Jiangsu, China and the Ethics Committee of Nanjing Agricultural University (NJAU.No20220520108).

## Author contributions

KL and QK: research idea, methodology, visualization, and supervision. XC, QK, XZ, CZ, PH, and HL: reagents, materials, and analysis tools. KL: writing—original draft and preparation. MK, ZB, II, HA, QS, and KL: writing—review and editing. All authors contributed to the article and approved the submitted version.

## Footnotes

1
https://github.com/QIIME2/q2-feature-classifier

2
http://www.arb-silva.de

3
https://yulab-smu.github.io/treedata-book

4
https://github.com/marbl/Krona/wiki

5
https://en.wikipedia.org/wiki/Venn_diagram

6
https://metacyc.org/

7
https://www.kegg.jp/
